# The case for altruism in institutional diagnostic testing

**DOI:** 10.1038/s41598-021-02605-4

**Published:** 2022-02-03

**Authors:** Ivan Specht, Kian Sani, Yolanda Botti-Lodovico, Michael Hughes, Kristin Heumann, Amy Bronson, John Marshall, Emily Baron, Eric Parrie, Olivia Glennon, Ben Fry, Andrés Colubri, Pardis C. Sabeti

**Affiliations:** 1 The Broad Institute of MIT and Harvard, Cambridge, MA 02142 USA; 2 Harvard College, Faculty of Arts and Sciences, Harvard University, Cambridge, MA 02138 USA; 3 FAS Center for Systems Biology, Department of Organismic and Evolutionary Biology, Faculty of Arts and Sciences, Harvard University, Cambridge, MA 02138 USA; 4 Howard Hughes Medical Institute, Chevy Chase, MD 20815 USA; 5 Colorado Mesa University, Grand Junction, CO 81501 USA; 6 COVIDCheck Colorado, Denver, CO 80202 USA; 7 Fathom Information Design, Boston, MA 02114 USA; 8 Program in Bioinformatics and Integrative Biology, University of Massachusetts Chan Medical School, Worcester, MA 01655 USA; 9 Massachusetts Consortium on Pathogen Readiness, Harvard Medical School, Harvard University, Boston, MA 02115 USA; 10 Department of Immunology and Infectious Diseases, Harvard T.H. Chan School of Public Health, Harvard University, Boston, MA 02115 USA

**Keywords:** Infectious diseases, Computational biology and bioinformatics, Computational models, Health policy

## Abstract

Amid COVID-19, many institutions deployed vast resources to test their members regularly for safe reopening. This self-focused approach, however, not only overlooks surrounding communities but also remains blind to community transmission that could breach the institution. To test the relative merits of a more altruistic strategy, we built an epidemiological model that assesses the differential impact on case counts when institutions instead allocate a proportion of their tests to members’ close contacts in the larger community. We found that testing outside the institution benefits the institution in all plausible circumstances, with the optimal proportion of tests to use externally landing at 45% under baseline model parameters. Our results were robust to local prevalence, secondary attack rate, testing capacity, and contact reporting level, yielding a range of optimal community testing proportions from 18 to 58%. The model performed best under the assumption that community contacts are known to the institution; however, it still demonstrated a significant benefit even without complete knowledge of the contact network.

## Introduction

During any societal crisis, altruism—defined as the “devotion to the welfare of others, regard for others, as a principle of action”—has the potential to both satisfy moral duty and maximize “utility,” leading to the best possible outcome for the greatest number of people^1^. It gains newfound urgency and utility during a pandemic, when important decisions must be made around allocating scarce resources, such as tests, therapies, and vaccines. In these instances more than ever, our own interests—our health, safety, and well-being—become highly interdependent on those of others. Specifically for communicable diseases, testing is patently a public good because a positive result can reduce others’ exposure and suffering by guiding isolation and quarantine practices.

Considerations of altruism and its efficacy have resurfaced in various COVID-19 response plans worldwide. As the disease began to spread in the U.S., it forced schools and businesses to cease in-person operations to mitigate its spread. To reopen, many of these institutions rushed to test their own members, enacting several-times-per-week or even daily testing protocols costing millions of dollars in hopes of preventing outbreaks^2^. Meanwhile, communities surrounding these institutions continued to struggle with ongoing clinical testing shortages and long delays for results. Even for institutional testing programs that considered supporting community testing, legal and regulatory barriers served as an additional deterrent from doing so.

To turn inward is a common and understandable approach during any crisis, but these expensive self-focused testing programs still left institutions blind to community cases that could potentially enter and spread like wildfire. For example, the NFL spent tens of millions in total throughout the Fall 2020 season on 623,000 tests, administered daily, for around 11,400 institutional members^3^. Yet the League still experienced outbreaks. They were not alone; outbreaks occurred within many similar testing programs, as the world witnessed prominently at the White House in Fall 2020.

We are now faced with the question of whether the confined use of significant resources to enable high-frequency testing within individual institutions alone is the most appropriate or effective way to contain a virus. We hypothesized that if institutions test altruistically—that is, designate a substantial portion of their testing capacity outside the institutions—it would not only be good for their communities, but also for them. That is, there would be lower case counts in these institutions themselves had their programs incorporated the testing of close contacts of its members into its testing strategy, thereby detecting potential COVID-19 encroachment.

This paper seeks to ascertain whether a self-focused or an altruistic testing approach is a more effective mitigation strategy. We provide a simple yet plausible epidemiological model to answer this question, comparing results under varying local community prevalence levels, social mitigation efforts, testing capacity, contact tracing adoption, and other parameters. We then discuss the significant real-world implications of our findings concerning how institutions might better allocate their available testing capacity.

## Epidemiological model

To test our central claim, we built an agent-based epidemiological model of a hypothetical institution such as a workplace, school, or similar organization, accounting for interactions within the institution as well as between the institution and its surrounding community. For a full, mathematically rigorous methods section, see Appendix A; here, we provide an intuitive, high-level description. We modeled two distinct groups: (1) institution members and (2) all of their first-degree close contacts outside the institution (hereafter referred to as the ‘periphery’). We assumed that the periphery remains unchanged throughout the simulation. We then assessed the effect of redistributing some of the institution’s testing capacity to the periphery, assuming for simplicity that diagnostic testing in the community was negligibly rare before the institution’s intervention. The model provided critical insight into the optimal proportion of tests to redistribute, given several baseline assumptions about viral dynamics, prevalence, and more. Moreover, we assumed no knowledge of the institution and the periphery beyond what institutional administrators/health officers might reasonably gather, such as the number of individuals, their frequent contacts, and the number of tests conducted. As such, our model gives a general framework by which institutions may assess possible testing protocols’ effectiveness.

Modeling viral propagation between an institution and its periphery requires detailed information on how the agents involved interact with each other. A typical means to capture this information is a simple undirected graph, in which two nodes (i.e., people) share an edge if and only if those two people interact during the modeled period^4^. In a real-world context, we might construct this graph by, for example, surveying members of the institution and its surrounding community about their social interactions. For our model, however, we assumed knowledge only of the mean and variance in contact numbers inside and outside the institution, which is likely more feasible to estimate in most contexts.

**Figure 1:**
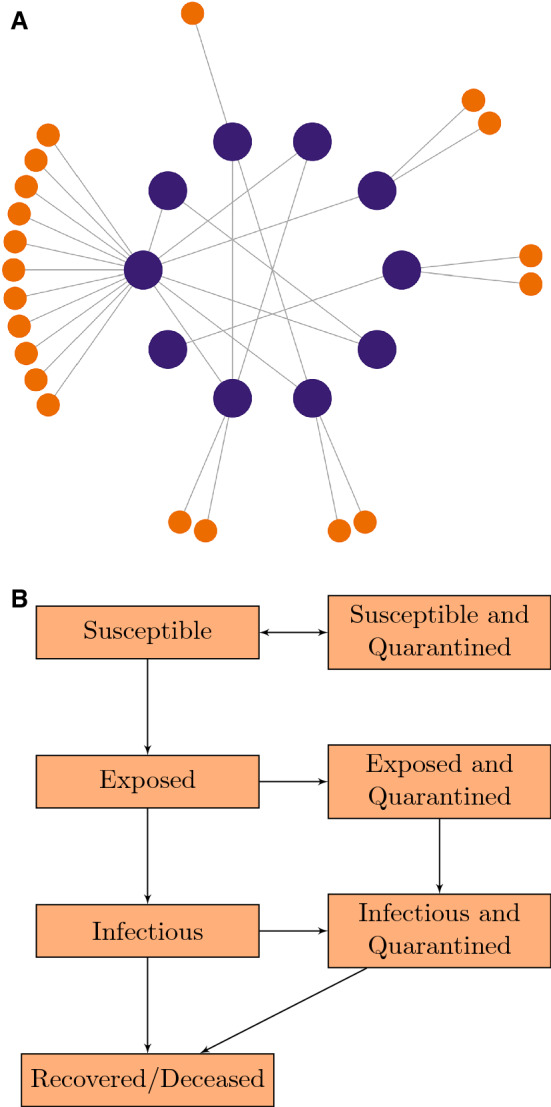
(**A**) Example of a contact network representing members of the institution (large, purple nodes) and their contacts in the periphery (small, orange nodes). Here we have 10 institution members who make an average of 2 contacts within the institution and 2 contacts outside the institution ($\text {variance} = 3$ for both distributions). (**B**) Flowchart of compartments and possible state transitions.

We proceeded by modeling *N* agents who interact according to a random graph, in which node degrees follow a Negative Binomial distribution fit to the observed mean and variance (see Appendix A for the random graph generation algorithm). We selected this distribution to reflect the overdispersed nature of both numbers of social contacts and numbers of onward transmissions per individual^5,6^. Within the institution, we assumed that agents mixed proportionately, meaning that the probability of any two nodes having an edge between them was proportional to the product of the node degrees. We chose to generate our graph for the institution using proportionate mixing, as opposed to one of the typical paradigms for random graph generation (Erdös-Rényi, Watts-Strogatz) in order to capture the overdispersion present in student social contact networks. To account for institution-periphery interactions, we assigned each agent a number of regular contacts made outside the institution, drawn from a different Negative Binomial distribution. See Fig. 1A for an example of a contact network generated via our methodology. This contact network may or may not be fully known to the institution; we included a model parameter that captures the extent to which agents report their contacts. This random graph constitutes the only stochastic portion of the model.

Having established a graphical contact network, we experimented with different testing strategies to simulate the spread of COVID-19. As a first step, we assumed that the periphery exhibits an epidemiological steady-state, computed based on the proportion of tests distributed there. By steady-state, we mean that the probability of an individual in the periphery being in a certain epidemiological state remains constant over the course of the simulation (see Appendix C for a more detailed treatment of this assumption). Within the institution, by contrast, we set the initial infection rate low in comparison to the periphery, reflective of the fact that many institutions returning to in-person activities have rigorous testing/quarantining protocols. For these agents, we modeled viral states probabilistically via an adapted *N*-intertwined mean-field approximation (NIMFA). In its original form, the NIMFA states that at a given time, the rate of transmission from agent *j* to adjacent agent *i* is proportional to the product of the marginal probabilities that *i* is susceptible and that *j* is infectious^7,8^. This approach offers the flexibility to encompass a wide range of epidemiological compartments while capturing the granularity of graphical contact networks^9–11^. NIMFA tends to overestimate viral propagation slightly as compared with fully stochastic methods, but these differences grow minimal with higher reproductive numbers as seen with COVID-19^8^. We opted for this method as opposed to a standard deterministic SEIR (susceptible-exposed-infectious-recovered) compartmental model to capture some of the observed differences in interaction levels between intra-institutional and extra-institutional contacts, as well as to capture variance in social interaction levels among institution members. More generally, it has been shown that mean-field approximation can reproduce expected epidemiological dynamics accurately for a wide range of contact degrees, without the need to compute an entire empirical distribution for the epidemic trajectory^12,13^.

We then extended NIMFA to include testing-based interventions. First, we allowed the outgoing transmission rate to vary between agents as a partial means of modeling overdispersion (with the rest coming from the node degree distribution). To account for quarantine compartments, we set the time-dependent rate at which agent *j* enters quarantine proportional to the product of the marginal probabilities that *j* is infectious and that *j* receives a test. In turn, this latter probability depended on the test distribution strategy, the probability that *j* had not previously tested positive, and the number of adjacent nodes in *j*’s contact network. Finally, accounting for the fact that COVID-19 cases exhibit an exposed (but not yet infectious) stage, we arrived at a detailed compartmental model that captures the epidemiological states of all agents, which l transition between states as depicted in Fig. 1B.

### Ethics statement

The study received a “Not Human Subjects Research” determination by the Broad Institute’s Office of Research Subject Protection.

## Results

We first applied our model to a mid-sized university (*N* = 10,000), using real data we gathered at Colorado Mesa University (CMU). CMU established a testing program in summer 2020 initially focused on university students and staff, and began supporting testing in the greater Mesa County community later that year. Contact tracers determined that the mean and variance of the number of close contacts within the institution were 2.3 and 2.4, respectively, and outside the institution were 0.2 and 1.9, respectively. They also found that the prevalence on campus at the beginning of the Spring 2020 semester was approximately 1%, and that they planned to conduct about 0.12 diagnostic tests per day per person. Supplementing our own data collection with that of the local public health authority, we compiled a complete set of parameters specific to CMU and ran the model accordingly (see Appendix D).

**Figure 2:**
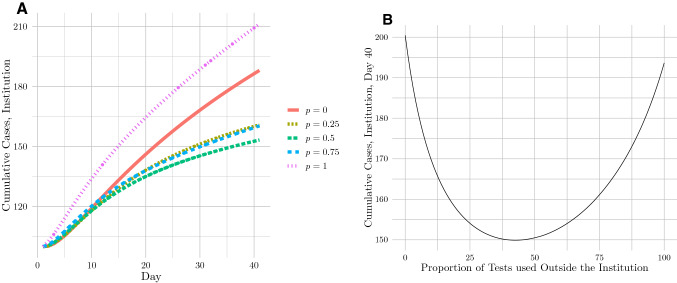
(**A**) Modeled cumulative cases over time at CMU under 5 different proportions *p* of peripheral testing; (**B**) cumulative cases on day 40 as a function of the proportion of tests deployed to the periphery, with the minimum at 45% peripheral testing.

Our initial model results based on the CMU parameters provided strong evidence in support of an altruistic testing strategy. We observed that the projected number of cases 40 days after the beginning of the modeled period was lowest when CMU deployed 45% of its tests to the periphery (see Fig. 2). This strategy reduced the institutional case count by 25% as compared to a self-focused testing strategy (i.e. 0% peripheral testing). However, our data from CMU—which informed our baseline parameters—were likely subject to several biases. While CMU administrators attested that they believed the data to represent the student body fairly well, students who contracted the virus (as every individual in our dataset did) were likely to have higher degrees of social interaction than those who did not, leading to a positive bias. On the other hand, CMU informed us that certain close contacts were likely not reported or otherwise not included in some cases, introducing a negative bias.

**Figure 3:**
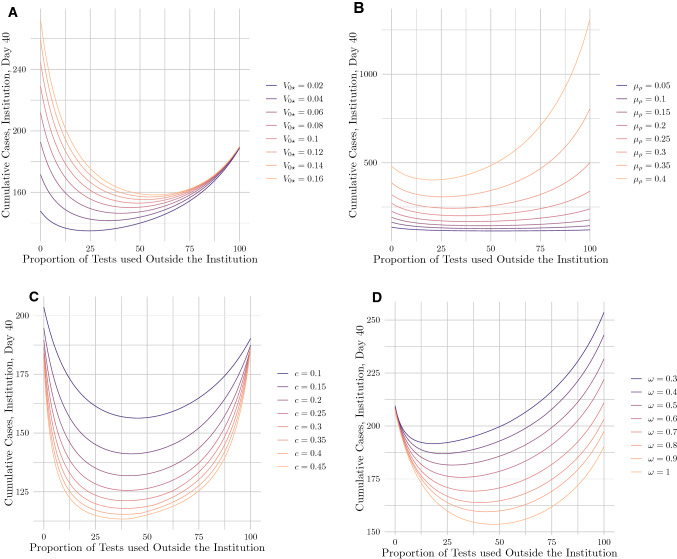
Cumulative cases on day 40 as a function of the proportion of tests deployed to the periphery under different values of (**A**) the initial prevalence in the periphery, $V_{0*}$; (**B**) the secondary attack rate among institution members, $\mu _\rho$; (**C**) the tests-per-person-per-day ratio, *c*, and (**D**) the proportion of contacts traced $\omega$.

Because of the potential limitations and biases of our CMU data, and because our model relies on numerous parameters that vary widely between institutions, we proceeded by demonstrating robustness to and characterizing the influence of several factors on our results. These included four key factors: local community prevalence, social mitigation efforts, testing capacity, and contact tracing adoption (see Fig. 3). Of course, there are more factors to assess, including distributions in numbers of contacts, variance in transmissibility, and initial prevalence within the institution. For an assessment of these factors, see Appendix B, or, for an interactive sensitivity analysis, visit https://ispecht.shinyapps.io/covid19-altruistic-testing/.

We first assessed sensitivity to the prevalence of COVID-19 among members of the periphery ($V_{0*}$). While the 6% positivity rate in Mesa County in January 2020 was reflective of national statistics at the time, different institutions faced significantly higher or lower caseloads in their surrounding communities, representative of factors such as density and public health resources that varied from place to place. Beyond that, rates have naturally varied over the course of the outbreak. We observed that under higher values of $V_{0*}$, the effectiveness of redistributing tests to the periphery grew, with the optimal proportion increasing from 24% when $V_{0*}$ = 2% to 58% when $V_{0*}$ = 16% (see Fig. 3A). This finding is unsurprising, given that the probability of any individual test detecting a case in the periphery grew with higher values of $V_{0*}$. In turn, the resulting quarantine (of both the peripheral member and their contact in the institution) minimized the probability of the virus breaching the institution.

Next, we turned to social mitigation efforts. While several model parameters capture these efforts, we focused on the secondary attack rate (SAR) for institution members–that is, the probability of transmission between an infectious individual and a susceptible one. Individuals may decrease the proportion of contacts they infect by, for example, wearing masks and socially distancing–thereby decreasing the observed SAR. Many institutions employ different mitigation measures, but altruism proved an effective strategy under a wide range of values for the observed SAR among institution members, denoted $\mu _\rho$ (see Fig. 3B). For $\mu _\rho$ = 0.05, the optimal proportion of peripheral testing lay at 54%; this proportion decreased to 25% when $\mu _\rho$ = 0.4. This again fell in line with our expectations, as for higher $\mu _\rho$ values, a single case within the institution had much greater potential to spread, limiting the effectiveness of peripheral testing and lowering the optimal proportion of tests to be administered outside the institution. On the other hand, when $\mu _\rho$ was low, a single case within the institution likely did not give rise to an outbreak. This made institutional testing less essential, leaving more capacity to establish a ‘barrier of defense’ in the periphery to prevent cases from breaching the institution in the first place.

We then focused on *c*, the number of tests administered by the institution per day per person. An intuitive way to think about this parameter is that on average, an institution member receives a diagnostic test every 1/*c* days. We found that test redistribution to the periphery remained an effective strategy even for relatively low values of *c*, such as *c* = 10% (see Fig. 3C). The optimal proportion of peripheral testing stayed relatively constant, ranging from about 38% to 48% for values of *c* between 45 and 10%. This result reflected the fact that under our baseline CMU parameters, institution members average about 10 times more contacts within the institution than outside it. As such, the size of the periphery was small, limiting the possible pathways for the virus to breach the institution. Our results tell us that even with limited testing resources, tests would best be used to prevent the virus from entering the institution via these pathways. Note that we also investigated different distributions of contacts within and outside the institution; for an analysis, see Appendix B, Fig. S1A–D.

Finally, we accounted for the fact that institution members may not report all of their contacts in the periphery, or may have contacts that the institution cannot test due to factors such as geographic disparity. We captured the proportion of reported, testable contacts with the parameter $\omega$. As our results suggest, contact reporting needed not be perfect for peripheral testing to help curb viral spread (see Fig. 3D). Even if institution members reported only 30% of their contacts, the optimal proportion of peripheral testing lay at 18%; this proportion increases to 45% as the fraction of reported contacts grows to 100%. This result makes sense because the most socially-active–and therefore riskiest–members of the institution had many peripheral contacts, at least some of which will likely be known to the institution even under imperfect contact tracing (e.g. familial contacts). A positive test from even just one of these contacts would send the original institution member into quarantine, allowing the ‘barrier of defense’ strategy to remain an effective means of protecting the institution.

We note that the model exhibited some slight variance between runs due to the stochastic nature of the contact network generation step. While such stochasticity slightly affected numerical values for case counts between model runs, the shapes of the curves in Figs. 2, 3 remained consistent.

## Discussion

Our model supports our hypothesis that the altruistic approach–in which institutions test beyond their walls–is the most effective protection strategy. In every instantiation of the model, we observed that deploying some proportion of diagnostic tests to the periphery significantly reduces the cumulative caseload at the end of a 40-day period. The optimal proportion of peripheral testing was 45% under baseline parameters and ranged from 18 to 58% under different levels of local prevalence, social interaction, testing, and contact tracing.

Our methods serve as a general framework for modeling one specific population within the context of another, and we hope that further research may help refine the intricacies of such dynamics. We also hope our work provides justification for institutions to consider implementing an altruistic testing strategy, and for legal and regulatory bodies to create a path for them to do so. We encourage institutions to partner with local public health authorities to support testing or connect members of the periphery with the appropriate testing provider, as Colorado Mesa University and the University of California Davis have done. As more institutions in disparate communities adopt this approach, we expect its impact to scale approximately linearly, as each altruistic testing program has the potential to impact its respective community in similar ways. Additionally, we would like to note that our model can be adapted to understand the impact of other interventions such as vaccines.

Finally, we wish to further unpack our meaning of altruism in the context of this work. As defined in the introduction, we intend for altruism to refer to the act of doing good, with or without regard for the implicit or explicit cost in doing so. We appreciate that in some disciplines, such as evolutionary biology, altruism specifically refers to organismal acts that inherently have—at least, an initial—cost to the self. That said, we recognize that there is an initial cost to the institution (that will be subsidizing the cost of testing individuals not affiliated with the institution). As such, using resources to test outside groups (e.g., logistical and financial costs) meets any definition of altruism.

Above all, our modeling work underscores one central message: The institution—and the surrounding community—will be safer (i.e., decreased burden of COVID-19) if it tests both within and beyond its figurative institutional “walls.” Our results urge a fundamental rethinking of how institutions with substantial testing capacity approach safety amid outbreaks. Epidemics are one of those rare instances where a seemingly selfless approach is, in fact, the most self-serving: institutions must help test beyond their walls to stay safe within them.

## Supplementary Information

Supplementary Information.

## Data Availability

Data from CMU and code used to implement the model and generate Fig. 2A are available at https://github.com/broadinstitute/covid19-altruistic-testing.
