# Supplementary material for: The case for altruism in institutional diagnostic testing
Source: Sci Rep. 2022 Feb 3;12:1857. doi: 10.1038/s41598-021-02605-4 (PMC8813946; doi:10.1038/s41598-021-02605-4)
Supplement: Supplementary file 1 — Supplementary Information. [file 41598_2021_2605_MOESM1_ESM.pdf]

The Case for Altruism in Institutional Diagnostic Testing
Supplementary Materials

Ivan Specht[†*1,2], Kian Sani[†1,3], Yolanda Botti-Lodovico[1,4], Michael Hughes[5], Kristin Heumann[5], Amy Bronson[5], John Marshall[5], Emily Baron[6], Eric Parrie[6], Olivia Glennon[7], Ben Fry[7], Andrés Colubri[‡*1,8], and Pardis C. Sabeti[‡*1,2,3,9,10]

[1]The Broad Institute of MIT and Harvard, Cambridge, MA, 02142, USA.
[2]Harvard College, Faculty of Arts and Sciences, Harvard University, Cambridge, MA, 02138, USA.
[3]FAS Center for Systems Biology, Department of Organismic and Evolutionary Biology, Faculty of Arts and Sciences, Harvard University, Cambridge, MA, 02138, USA.
[4]Howard Hughes Medical Institute, Chevy Chase, MD, 20815, USA.
[5]Colorado Mesa University, Grand Junction, CO, 81501, USA.
[6]COVIDCheck Colorado, Denver, CO, 80202, USA.
[7]Fathom Information Design, Boston, MA, 02114, USA.
[8]Program in Bioinformatics and Integrative Biology, University of Massachusetts Medical School, Worcester, MA 01655, USA.
[9]Massachusetts Consortium on Pathogen Readiness, Harvard Medical School, Harvard University, Boston, MA, 02115, USA.
[10]Department of Immunology and Infectious Diseases, Harvard T.H. Chan School of Public Health, Harvard University, Boston, MA, 02115, USA.

[†]These authors contributed equally.
[‡]These authors jointly supervised this project.
[*]Corresponding authors: ispecht@broadinstitute.org, andres.colubri@umassmed.edu, pardis@broadinstitute.org

December 28, 2021

Appendices

A Methods

Overview. Here we derive the full set of modeling equations we used to conduct our analysis. We construct an agent-based epidemiological model (ABM) with known contact network in order to compare the effectiveness of various testing regimes at controlling viral spread within an institutional context. Specifically, we model two groups of agents—the members of an "institution" (such as a school, business, etc.) and all of their close contacts outside the institution (henceforth referred to as the "periphery")—and assume the institution has access to a disproportionately-large tests-per-person-per-day ratio in comparison to the periphery. We then analyze the effect of redistributing some of the institution's testing capacity to the periphery. The model provides key insight into the optimal proportion of tests to be redistributed in such a way. More broadly, the set of equations we provide serve as a general framework by which institutions may assess the relative effectiveness of various testing protocols.

Contact Network Generation. We simulate an institution with N agents that interact according to a random graph whose node degree distribution reflects known contact patterns of the institution's members. Let M_i be a random variable representing the number of edges connected to node i. To reflect the overdispersion typically associated with node degrees in social contact networks, we assume that the M_i's are i.i.d. negative binomial with mean μ and variance σ^2 [5]. We can express M_i as a function of the activity levels $\mathbf{a} = (a_1, \ldots, a_N)$ of the N individuals—parameters that represent sociability, whose distribution we will derive. Assuming proportionate mixing, we define ϵ_{ij}, the probability of nodes i and j sharing an edge, to be

$$\epsilon_{ij} = \frac{a_i a_j}{\sum_{k=1}^{N} a_k}$$

for $i \neq j$, and 0 for $i = j$. Assuming that ϵ_{ij} is small in general (an assumption we will justify below), we have that $X_i | \mathbf{A} = \mathbf{a} \dot{\sim} \text{Pois}(a_i)$, where $\dot{\sim}$ means "approximately distributed as." So, we now need to choose a distribution for the A_i's to ensure that the marginal distribution of X_i is Negative Binomial. Since the Negative Binomial distribution may be expressed as the Poisson distribution compounded with the Gamma distribution, we model the A_i's as i.i.d. Gamma(α, λ) random variables. To solve for the parameters, we have that

$$\mu = \text{E}[X_i] = \text{E}[\text{E}[X_i|\mathbf{A}]] = \text{E}[A_i] = \frac{\alpha}{\lambda}$$

$$\sigma^2 = \text{Var}[X_i] = \text{E}[\text{Var}[X_i|\mathbf{A}]] + \text{Var}[E[X_i|\mathbf{A}]] = \text{E}[A_i] + \text{Var}[A_i] = \frac{\alpha}{\lambda} + \frac{\alpha}{\lambda^2}.$$

Thus,

$$\alpha = \frac{\mu^2}{\sigma^2 - \mu} \quad \text{and} \quad \lambda = \frac{\mu}{\sigma^2 - \mu}.$$

Finally, the assumption that $a_i a_j \ll \sum_{k=1}^{N} a_k$ follows from the Cauchy-Schwarz inequality. We have that:

$$\text{E}[\epsilon_{ij}] = \text{E}\left[\frac{A_i A_j}{\sum_{k=1}^{N} A_k}\right] \leq \sqrt{\text{E}[A_i^2 A_j^2]\text{E}\left[\frac{1}{(\sum_{k=1}^{N} A_k)^2}\right]}.$$

For the first expectation within the radical, we have that

$$\mathrm{E}[A_i^2 A_j^2] = \mathrm{E}[A_i^2]\mathrm{E}[A_j^2] = \mathrm{E}[A_i^2]^2 = \frac{\alpha^2(1+\alpha)^2}{\lambda^4}.$$

For the second expectation, let $S = \sum_{k=1}^{N} A_k$. Then $S \sim \text{Gamma}(N\alpha, \lambda)$. Therefore

$$\mathrm{E}\left[\frac{1}{S^2}\right] = \int_0^\infty \frac{f_S(s)}{s^2}\,\mathrm{d}s = \frac{\lambda^2}{(N\alpha-1)(N\alpha-2)}.$$

so long as $N\alpha > 2$. Thus,

$$\mathrm{E}[\epsilon_{ij}] \le \sqrt{\frac{\alpha^2(1+\alpha)^2}{\lambda^2(N\alpha-1)(N\alpha-2)}} < \sqrt{\frac{\alpha^2(1+\alpha)^2}{\lambda^2(N\alpha-2)^2}} = \frac{\alpha(1+\alpha)}{\lambda(N\alpha-2)}$$

which approaches 0 as $N \to \infty$.

Thus, we may generate our contact network by drawing the a_i's from the Gamma(α, λ) distribution, then drawing a Bern(ϵ_{ij}) random variable for each unordered pair of nodes $\{i, j\}$ to generate the edges of the graph stochastically. Note that the probability of ϵ_{ij} exceeding 1 is small because $P(\epsilon_{ij} \ge 1) \le \mathrm{E}[\epsilon_{ij}]$ by Markov's inequality; in practice, this never occurred for all parameter combinations studied in this paper.

For contacts outside the insitution, we assume for simplicity that no two agents share a peripheral contact. Letting Y_i be the number of close contacts in the network of agent i outside the institution, we model the Y_i's as i.i.d. Negative Binomial random variables with mean μ_* and variance σ_*^2. Contact reporting outside the institution, however, is likely imperfect—and even among reported contacts, some may be unwilling to receive a diagnostic test. To address this, we introduce the parameter ω, which represents the probability of any given contact outside the institution being traced and testable by the institution. Then, letting Z_i be the number of traced and testable contacts made by agent i outside the institution, we model $Z_i | Y_i \sim \text{Bin}(Y_i, \omega)$. Moving forward, we will treat the contact network as fixed, and thus we will use the notations $\mathbf{x}, \mathbf{y}$, and $\mathbf{z}$ to represent the values taken on by random variables $\mathbf{X}, \mathbf{Y}$, and $\mathbf{Z}$. We refer to the set of all traced and testable people outside the institution as the "known periphery"

Viral propagation model. To capture the structure of a given graphical contact network, we take an agent-based approach for members of the institution; for the periphery, we implement a compartmental model that describes the population at large. Moreover, we assume that the prevalence in the periphery exhibits an epidemiological steady-state, a somewhat atypical assumption that we will explain further below and test as part of our sensitivity analysis (see Figure 3A in the main text and Appendices B-C). Finally, we include inter-compartmental "flux" terms in our modeling equations to account for the propagation of cases from the periphery into the institution.

We begin with the compartmental model for the periphery. We model five compartments: Susceptible (S_*), Exposed (E_*), Infectious (V_*), Exposed/Infectious and Quarantined (Q_*), and Recovered/Deceased (R_*). Note that from now on, we will use the lower star notation for variables/parameters specific to the periphery. For our purposes we take exposed to mean "contracted the virus, but not yet infectious," and assume that every agent enters the exposed stage before entering the infectious stage [16]. Let γ and δ be the recovery rate and exposed-to-infectious transition rates, respectively. Let c be the tests-per-person-per-day ratio, of which a proportion p are designated for members of the known periphery. Assuming for simplicity that each member of the

known periphery is equally likely to be tested on any given day, we may set η_*, the daily probability of an agent in the known periphery being tested, as follows:

$$\eta_* = \frac{pcN}{\sum_{i=1}^{N} z_i}. \tag{1}$$

We only need to compute the steady-state probabilities of being exposed and infectious in the periphery, since the other states do not substantively impact our model. We assume that *prior to interventions*, i.e. testing and quarantine, the steady-state probability of being infectious is V_{0*}. Given the transition rates, we may then assume that E_{0*}, the steady-state probability of being exposed prior to interventions, is $\gamma V_{0*}/\delta$. Finally, since γV_{0*} recover per day, it follows that γV_{0*} must transition from susceptible to infectious each day to maintain the steady state.

Next, we include the effects of interventions, which affect only the known periphery. COVID-19 diagnostic tests have been shown to be highly reliable during the infectious stage of illness, but often fail to detect the extremely low viral loads present during the exposed stage [16, 17]. As such, we let ψ_E be the test sensitivity during the exposed stage and ψ_V be the test sensitivity during the infectious stage. Modeling the exposed-to-quarantined and infectious-to-quarantined rates as $\eta_*\psi_E$ and $\eta_*\psi_V$, respectively, we arrive at the following system of differential equations:

$$\frac{\mathrm{d}E_*(t)}{\mathrm{d}t} = -\delta E_*(t) - \eta_*\psi_E E_*(t) + \gamma V_{0*} \tag{2}$$

$$\frac{\mathrm{d}V_*(t)}{\mathrm{d}t} = -\gamma V_*(t) - \eta_*\psi_V V_*(t) + \delta E_*(t) \tag{3}$$

where $E_*(t)$ and $V_*(t)$ are the probabilities of an individual in the known periphery being exposed and infectious at time t, respectively. Setting both derivatives equal to 0 and solving in terms of V_{0*}, we obtain the steady states, which we denote $E_{\infty*}$ and $V_{\infty*}$, respectively:

$$E_{\infty*} = \frac{\gamma V_{0*}}{\eta_*\psi_E + \delta} \tag{4}$$

$$V_{\infty*} = \frac{\gamma\delta V_{0*}}{(\eta_*\psi_V + \gamma)(\eta_*\psi_E + \delta)} \tag{5}$$

In the "unknown periphery" (i.e. members of the periphery who institution members do not report), we may assume that the the probabilities of being exposed and infectious are E_{0*} and V_{0*}, respectively.

We now turn our focus to the institution. Given the level of granularity provided by contact networks, along with the difficulty in capturing agent-specific conditions through more traditional epidemiological methods (i.e. compartmental modeling for the population at large), we model the state of each agent in the institution probabilistically using N-Intertwined Mean-Field Approximation (NIMFA) [7]. A widely-used and computationally-efficient approximation for the true stochastic process, the NIMFA approximates the joint probability of some agent i being susceptible and some other agent j being infectious as the product of the marginals. In its original form, the NIMFA states that:

$$\frac{\mathrm{d}v_i(t)}{\mathrm{d}t} = -\gamma v_i(t) + s_i(t)\sum_{j=1}^{N} \mathbb{1}_{ij}\beta_j v_j(t) \tag{6}$$

where $v_i(t)$ and $s_i(t)$ are the probabilities of agent i being infectious and susceptible at time t, respectively; $\mathbb{1}_{ij}$ is the indicator of an edge between i and j; γ is the recovery rate (as before), and β_j is the probability of transmission between infectious agent j and a susceptible agent per unit time.

We allow for heterogeneity in $\boldsymbol{\beta} = (\beta_1, \ldots, \beta_M)$, where M is the total number of agents in both the institution and the periphery, to account for part of the overdispersion in COVID-19 transmission (the other part coming the node degree distribution in our graphical model). To derive $\boldsymbol{\beta}$, we begin by drawing $\boldsymbol{\rho} = (\rho_1, \ldots, \rho_M)$, the (heterogeneous) secondary attack rate specific each agent, from a Beta distribution with mean μ_ρ and variance σ_ρ^2. Since we defined ρ_i to be the probability of transmission over the course of agent i's infection, and since the probability density of recovery at time t is equal to $\gamma \exp(-\gamma t)$, we may write the total probability of transmission ρ_i in terms of the daily probability of transmission β_i:

$$\rho_i = \int_0^\infty (1 - (1 - \beta_i)^t)\gamma \exp(-\gamma t)\,\mathrm{d}t. \tag{7}$$

And, solving for β_i, we obtain:

$$\beta_i = 1 - \exp\left(-\frac{\gamma \rho_i}{1 - \rho_i}\right). \tag{8}$$

Note that in the case of COVID-19, limited literature exists on the variation in the secondary attack rate between agents, and the extent to which this phenomenon influences overdispersion is not known. As such, our distribution of the ρ_i's will be weakly informed, so we assess sensitivity to μ_ρ and σ_ρ^2 in Appendix B.

Having solved for $\boldsymbol{\beta}$, we now adapt the NIMFA to include additional transmission pathways and compartments for each individual agent while staying true to the original idea. Within the institution, we model seven compartments: Susceptible (S), Exposed (E), Infectious (V), Recovered/Deceased (R), Susceptible and Quarantined (U), Exposed and Quarantined (W), and Infectious and Quarantined (Q). See Figure 1B (main text) for a diagram of all possible state transitions. For any state $Z \in \{S, E, V, R, U, W, Q\}$, we use the notation $z_i(t)$ to mean the probability that individual i is in state Z at time t. Since our agent-based model applies only to members of the institution, we allow i to range over integers only from 1 to N, inclusive. Revising (6) to account for the "exposed" compartment and for each agent's outside-the-institution contacts, and then applying a Poisson approximation (to avoid the fact that the sum of the transmission probabilities may exceed 1), we obtain:

$$\frac{\mathrm{d}e_i(t)}{\mathrm{d}t} = -\delta e_i(t) + s_i(t)\left(1 - \exp\left(-\sum_{j=1}^{M} \mathbb{1}_{ij}\beta_j v_j(t)\right)\right) \tag{9}$$

$$\frac{\mathrm{d}v_i(t)}{\mathrm{d}t} = -\gamma v_i(t) + \delta e_i(t). \tag{10}$$

Note that for $j > N$, we take $v_j(t)$ to equal $V_{\infty *}$ if j is a member of the known periphery and V_{0*}, otherwise. For ease of notation, from now on we let

$$\mathrm{B}_i(t) = \left(1 - \exp\left(-\sum_{j=1}^{M} \mathbb{1}_{ij}\beta_j v_j(t)\right)\right).$$

Next, we revise (9) and (10) to include the impact of quarantine due to a positive test result. To do so, we first need to decide on a prioritization for diagnostic tests within the institution. Let $\eta_i(t)$ be the probability that agent i is tested at time t. It is natural that we model this probability as roughly proportional to the number of known close contacts agent i has (both within and outside the institution) times the probability that agent i has not already been identified as a positive case. However, we also need to satisfy that the number of tests administered within the institution per unit time always sums to $(1-p)cN$, and choosing a constant of proportionality to satisfy this condition may cause $\eta_i(t)$ to be greater than 1 for certain i. As such, we instead model $\eta_i(t)$ as

$$\eta_i(t) = 1 - \exp\Bigg(- \alpha(t)(x_i + z_i)(s_i(t) + e_i(t) + v_i(t))\Bigg) \tag{11}$$

with $\alpha(t)$ chosen to satisfy

$$\sum_{i=1}^{N} \eta_i(t) = (1-p)cN. \tag{12}$$

Note that no closed-form solution for $\alpha(t)$ exists; in practice, we compute it numerically for each discrete timestep Δt. Just as in the model for the periphery, we then set the exposed-to-quarantined and infectious-to-quarantined rates (due to a positive test result) equal to $\eta_i(t)\psi_E$ and $\eta_i(t)\psi_V$, respectively. Now accounting for the quarantine rate due to a positive test result, we obtain:

$$\frac{\mathrm{d}e_i(t)}{\mathrm{d}t} = -\delta e_i(t) - \eta_i(t)\psi_E e_i(t) + s_i(t)\mathrm{B}_i(t) \tag{13}$$
$$\frac{\mathrm{d}v_i(t)}{\mathrm{d}t} = -\gamma v_i(t) - \eta_i(t)\psi_V v_i(t) + \delta e_i(t) \tag{14}$$
$$\frac{\mathrm{d}w_i(t)}{\mathrm{d}t} = -\delta w_i(t) + \eta_i(t)\psi_E e_i(t) \tag{15}$$
$$\frac{\mathrm{d}q_i(t)}{\mathrm{d}t} = -\gamma q_i(t) + \eta_i(t)\psi_V v_i(t) + \delta w_i(t). \tag{16}$$

Finally, we include the effect of quarantine due to a positive test result from an agent's first-degree contact. Again applying Poisson approximation to the events of each institutional agent's first-degree contacts testing positive, we may write the time-dependent rate $\Gamma_i(t)$ at which agent i enters quarantine due to a known neighbor testing positive as:

$$\Gamma_i(t) = \left(1 - \exp\left(-\eta_* z_i(\psi_E E_{\infty *} + \psi_V V_{\infty *}) - \sum_{j=1}^{N} \mathbb{1}_{ij}\eta_j(t)(\psi_E e_j(t) + \psi_V v_j(t))\right)\right).$$

Revising (13-16) once again and adding in the remaining states, we arrive at our complete set of differential equations:

$$\frac{\mathrm{d}s_i(t)}{\mathrm{d}t} = -\, s_i(t)\Gamma_i(t) - s_i(t)\mathrm{B}_i(t) + \eta_i(t)u_i(t) \tag{17}$$
$$\frac{\mathrm{d}e_i(t)}{\mathrm{d}t} = -\, e_i(t)\Gamma_i(t) - \delta e_i(t) - \eta_i(t)\psi_E e_i(t) + s_i(t)\mathrm{B}_i(t) \tag{18}$$
$$\frac{\mathrm{d}v_i(t)}{\mathrm{d}t} = -\, v_i(t)\Gamma_i(t) - \gamma v_i(t) - \eta_i(t)\psi_V v_i(t) + \delta e_i(t) \tag{19}$$
$$\frac{\mathrm{d}u_i(t)}{\mathrm{d}t} = -\, \eta_i(t)u_i(t) + s_i(t)\Gamma_i(t) \tag{20}$$

$$\frac{\mathrm{d}w_i(t)}{\mathrm{d}t} = -\,\delta w_i(t) + e_i(t)\Gamma_i(t) + \eta_i(t)\psi_E e_i(t) \tag{21}$$

$$\frac{\mathrm{d}q_i(t)}{\mathrm{d}t} = -\,\gamma q_i(t) + v_i(t)\Gamma_i(t) + \eta_i(t)\psi_V v_i(t) + \delta w_i(t) \tag{22}$$

$$\frac{\mathrm{d}r_i(t)}{\mathrm{d}t} = \gamma v_i(t) + \gamma q_i(t). \tag{23}$$

For the analyses conducted in this paper, we solved this system of differential equations using a discrete timestep of $\Delta t = 1$ day.

B Further Sensitivity Analysis

Here we build on the analysis in the Results section by assessing sensitivity to μ, σ^2, μ_*, σ_*^2, σ_ρ^2, and $\mathbf{v}_0$, using a one-at-a-time approach. As in the Results section, for each parameter, we selected a range of possible values under which to plot cumulative cases on day 40 as a function of p (the proportion of tests deployed outside the institution). We found that the strategy of deploying at least some proportion of tests to the periphery is robust to a wide variety of parameter combinations (see Figure S1). Note that we observed some noise in our analyses of μ_* and σ_*^2 due to the skewness in the distribution of peripheral contacts; nonetheless, we still observed a clear trend in the day-40 cumulative case count as a function of peripheral testing under each value of μ_* and σ_*^2 (see Figures S1C, S1D).

C Peripheral Steady State

In our epidemiological model, we made the critical assumption that the periphery exhibits an epidemiological steady state. The rationale behind this assumption is that over the course of the COVID-19 pandemic, overall trends in cases (at the county, state, and national levels alike) have fluctuated based on a number of factors outside the control of individual institutions. As such, we chose not to model any change in the extra-institutional prevalence, but instead assumed it to be constant and then assess sensitivity to this factor (see Figure 3A, main text). Here we provide additional analysis of how long after the implementation of testing in the periphery it takes in order to achieve such a steady state. Numerically approximating $E_*(t)$ and $V_*(t)$ based on equations (2-3), we obtained the curves shown in Figure S2, which provide estimates for the probabilities of being exposed and infectious in the periphery as a function of the duration of testing. We set all other relevant parameters to their baseline values (see Appendix D).

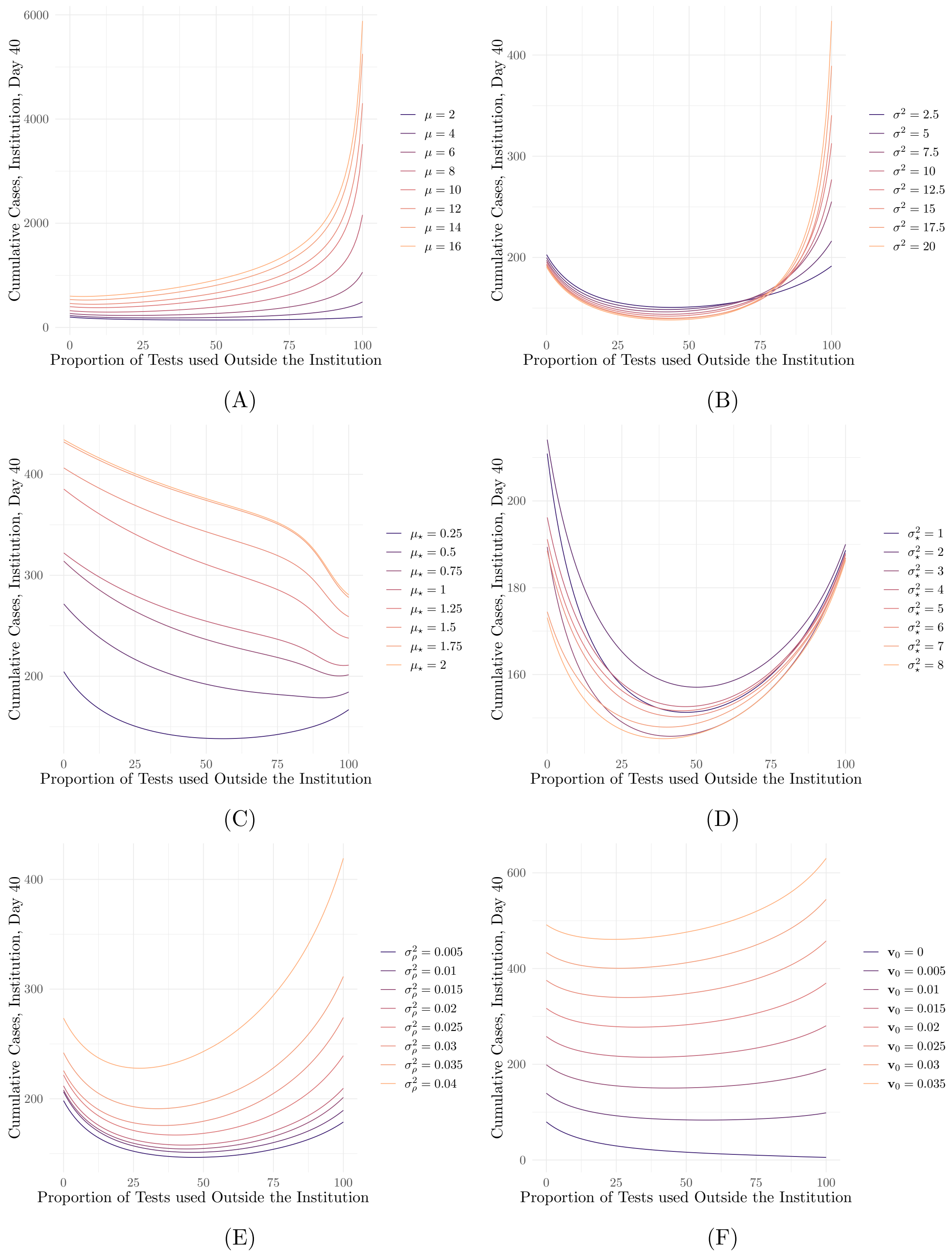

Figure S1: Cumulative cases on day 40 as a function of the proportion of tests deployed to the periphery under different values of (A) the mean number of institutional contacts, μ; (B) the variance in institutional contacts, σ^2; (C) the mean number of peripheral contacts, μ_*; (D) the variance in peripheral contacts, σ^2_*; (E) the variance in the secondary attack rate, σ^2_ρ; and (F) the initial prevalence at the institution, $\mathbf{v}_0$ (assumed to be uniform among agents)

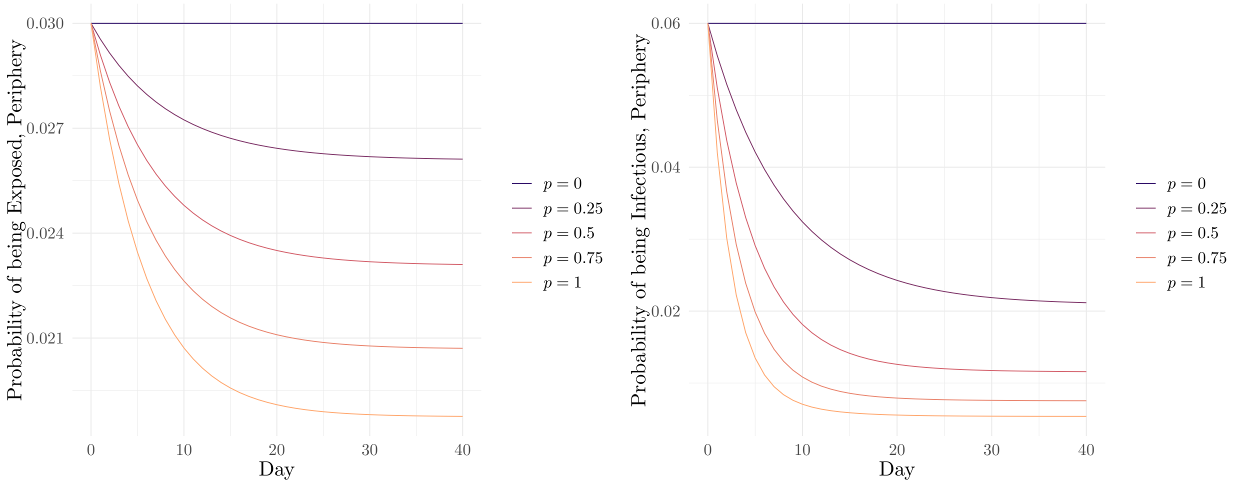

(A) (B)

Figure S2: Modeled probabilities of an agent in the periphery being (A) exposed and (B) infectious over time starting with the implementation of community testing at time 0, under 5 different proportions p of peripheral testing.

D Model Parameters

**Symbol**	**Description**	**Value**	**Citation**
N	Number of people in the institution	10,000	
μ	Mean number of contacts per agent at the institution	2.3	
σ^2	Variance in number of contacts per agent at the institution	2.4	
μ_*	Mean number of contacts per agent outside the institution	0.23	
σ_*^2	Variance in number of contacts per agent outside the institution	1.8	
ω	Proportion of contacts made outside the institution known to testing authorities	1	
μ_ρ	Mean secondary attack rate	0.16	[14]
σ_ρ^2	Variance in secondary attack rate among agents	0.01	$\star$
γ	Recovery rate	0.1	[15]
δ	Exposed-to-infectious transition rate	0.2	[16]
$\mathbf{v}_0$	Initial probability of infection within the institution, for each agent	0.01 (uniform)	
V_{0*}	Initial probability of infection outside the institution	0.06	
c	Number of tests per day per person	0.12	
ψ_E	Test sensitivity during the exposed stage	0.2	[17]
ψ_V	Test sensitivity during the infectious stage	1	[17]

Table S3: Baseline model parameters, explanations, values, and citations. Parameters with no citation are estimated based on data gathered at CMU. Parameters marked $\star$ are weakly-informed.
